# Solutions of a Class of Switch Dynamical Systems

**DOI:** 10.3390/e27020158

**Published:** 2025-02-02

**Authors:** Marius-F. Danca

**Affiliations:** STAR-UBB Institute, Babes-Bolyai University, 400084 Cluj-Napoca, Romania; m.f.danca@gmail.com

**Keywords:** Filippov regularization, one-sided Lipschitz condition, strengthened one-sided Lipschitz condition, differential inclusion, approximate selection theorem

## Abstract

In this paper, the solutions of a class of switch dynamical systems are investigated. The right-hand side of the underlying equations is discontinuous with respect to the state variable. The discontinuity is represented by jump discontinuous functions such as signum or Heaviside functions. In this paper, a novel approach of the solutions of this class of discontinuous equations is presented. The initial value problem is restated as a differential inclusion via Filippov’s regularization, after which, via the approximate selection results, the differential inclusion is transformed into a continuous, single-valued differential equation. Besides its existence, a sufficient uniqueness condition, the strengthened one-sided Lipschitz Condition, is also introduced. The important issue of the numerical integration of this class of equations is addressed, emphasizing by examples the errors that could appear if the discontinuity problem is neglected. The example of a mechanical system, a preloaded compliance system, is considered along with other examples.

## 1. Introduction

Systems with a discontinuous right-hand side are ideal mathematical models describing physical phenomena whose time series data exhibit different dynamical modes. They can be found mainly in the theory of mechanical systems, where switch-type functions like sgn or Heaviside are used to describe phenomena. This kind of discontinuity can be found generally in two-dimensional mechanical systems: oscillating systems combined with dry and viscous damping, systems with dry friction, systems with stick and slip modes, forced vibrations, brake processes with locking phases, control synthesis for uncertain systems, elastoplasticity, and also in control theory, game theory, optimization, calculus of variations, biological systems, electrical circuits, complex networks, power electronics, etc. (see, e.g., [1,2] and references therein). Due to the switch determined by the discontinuity related to sgn-like functions, these systems are called “switch systems”.

There exist two main strategies with which to approach numerical switch systems: One is to ignore the discontinuities (“time stepping” methods) and to rely on a local error estimator such that the error remains acceptably small. The other strategy is to determine a scalar event function to define the discontinuity. The intersection point serves as the new starting point to continue the numerical solution (“event-driven” methods). To integrate numerically discontinuous Ordinary Differential Equations (ODEs), there exist dedicated numerical methods, most of them using the theory of differential inclusions (DIs) [3,4,5,6,7,8].

In this context, in this paper, a novel approach is proposed: restarting the discontinuous initial value problem (IVP), which models the switch system, into a continuous single-valued problem using the theory of differential inclusions, which can be numerically integrated using standard schemes for differential equations. Moreover, the inherent error problems of the numerical integration of these equations without managing the discontinuity is analyzed. Also, the strengthened one-sided Lipschitz condition, considered in the theory of differential inclusions, is adapted and proposed for the considered class of equations.

The manuscript is structured as follows: Section 2 presents the considered class of switch systems with some examples. Next, Section 3 contains a survey of some known results on general solutions, differential inclusions, and approximate selection and also presents the existence and uniqueness of solutions. In Section 4, some numerical simulations are considered. The Conclusion section ends the paper.

## 2. Switch Dynamical Systems

The class of piece-wise continuous (PWC) systems considered in this paper are time-continuous and discontinuous on the right-hand side, with the discontinuity concerning the state variable. The systems are modeled using the following autonomous initial value problem (IVP):

(1) $$\begin{array}{cc} \; & \dot{x}=f\left(x\right):=g\left(x\right)+As\left(x\right),\\ \; & x\left(0\right)=x_{0},\;t\in I=\left[0,T\right], \end{array}$$

where $s:R^{n}\to R^{n}$; $s\left(x\right)={\left(s\left(x_{1}\right),s\left(x_{2}\right),...,s\left(x_{n}\right)\right)}^{T}$ is a piece-wise continuous function considered in this paper with components $x_{i}\mapsto s\left(x_{i}\right)$, usually $\mathrm{sgn}\;(x_{i})$ or Heaviside $H\left(x_{i}\right)=\left(1+\mathrm{sgn}\left(x_{i}\right)\right)/2$. T∈R,T>0; and the real matrix $A\in R^{n\times n}$ is supposed to have at least one nonzero element.

The following assumption is considered:

**H1**  $g:R^{n}\to R^{n}$ is a Lipschitz continuous function.

Hereafter, for simplicity, unless necessary, the IVPs are written without the initial condition $x_{0}$.

Systems described by (1) represent a large class of switch dynamical systems.

*Example 1.*  The mechanical system studied in this paper is the preloaded compliance system with a single degree freedom [9] modeled by a second-order differential equation with an initial condition at x(0).

(2) $$m\ddot{y}+c\dot{y}+h\left(y\right)=P\cos\omega t,\;y\left(0\right)=y_{0},\;\dot{y}\left(0\right)=y_{1}.$$

**Figure 1:**
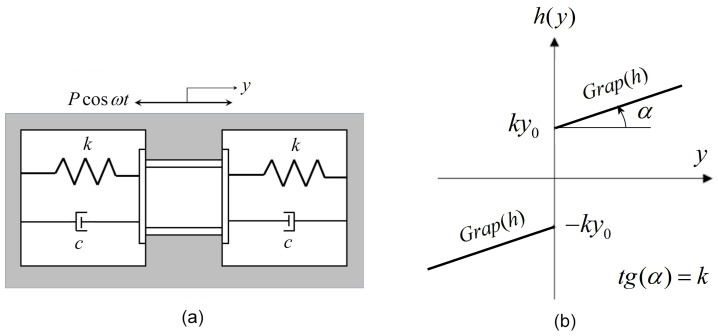
(**a**) Model of system (2); (**b**) graph of function *h* (4).

The system is excited by the periodic external force Pcosωt, *m* represents the mass of the system, *k* is the stiffness, and *c* is the viscous damping coefficient (Figure 1a). The restoring force *h* presents a discontinuity at y=0 (Figure 1b), with the jump having the value of $2ky_{0}$.

$$h\left(y\right)=\left\{\begin{array}{c} k(y+y_{0}),\;y\geq 0\\ k(y-y_{0}),\;y\leq 0 \end{array}\right.$$

where *k* represents the angular coefficient (slope) of the line $k(y\pm y_{0})$. The interpretation of the set-valued form (segment $\left[-ky_{0},ky_{0}\right]$) for function *h* will be analyzed in Section 3.

**Proposition 1.**  *System (2) can be modeled as IVP (1).*

**Proof.**  Consider the change in variable $x=y/y_{0}$ and $\tau =\omega_{n}t$. $p=P/kx_{0}$, and $\nu =\omega /\omega_{0}$. Then, the dimensionless form of system (2) becomes (see details in [9])

(3) $$\ddot{x}\left(\tau \right)+2\gamma \dot{x}\left(\tau \right)+h\left(x\left(\tau \right)\right)=p\cos\nu \tau ,$$

where h(x), after the mentioned substitutions, becomes

(4) $$h\left(x\right)=\left\{\begin{array}{c} x+1,\;y\geq 0,\\ x-1,\;y\leq 0, \end{array}\right.$$

or h(x)=x+sgn(x).

Therefore, the standard autonomous form of system (3) becomes

(5) $$\begin{array}{cc} {\dot{x}}_{1} & =x_{2},\\ {\dot{x}}_{2} & =-2\gamma x_{1}-x_{1}-\mathrm{sgn}\left(x_{1}\right)+p\cos\left(x_{3}\right),\\ {\dot{x}}_{3} & =\nu , \end{array}$$

with

$$f\left(x_{1},x_{2},x_{3}\right):=\left(\begin{array}{c} f_{1}\left(x_{1},x_{2},x_{3}\right)\\ f_{2}\left(x_{1},x_{2},x_{3}\right)\\ f_{3}\left(x_{1},x_{2},x_{3}\right) \end{array}\right)=\left\{\begin{array}{c} x_{2},\\ -2\gamma x_{1}-x_{1}-\mathrm{sgn}\left(x_{1}\right)+p\cos\left(x_{3}\right),\\ \nu . \end{array}\right.$$

Thus,

$$A=\left(\begin{array}{ccc} 0 & 0 & 0\\ -1 & 0 & 0\\ 0 & 0 & 0 \end{array}\right),\;g\left(x\right)=\left(\begin{array}{c} x_{2}\\ -2\gamma x_{1}-x_{1}+P\cos\left(x_{3}\right)\\ \nu \end{array}\right),$$

and

$$s\left(x\right)=\left(\begin{array}{c} \mathrm{sgn}(x_{1})\\ \mathrm{sgn}(x_{2})\\ \mathrm{sgn}(x_{3}) \end{array}\right).$$

□

**Figure 2:**
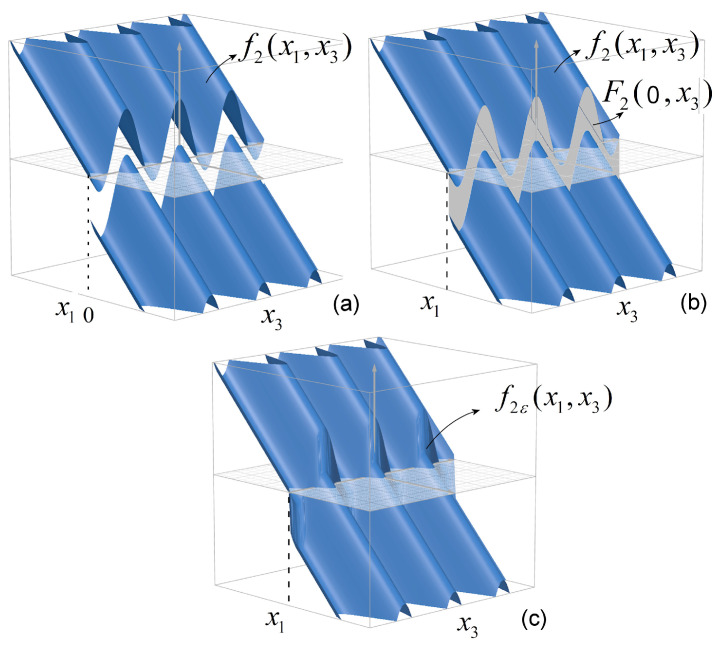
(**a**) Graph of the discontinuous component, $f_{2}$, of preloaded compliance system (5); (**b**) graph of set-valued function $F_{2}$; (**c**) graph of the approximate selection $f_{2\varepsilon }$.

The graph of $f_{2}$ is presented in Figure 2a.

*Example 2.*  A steam turbine control system [10] is modeled as follows:

(6) $$\begin{array}{cc} {\dot{x}}_{1} & =p(x_{3}-x_{1}-\mathrm{sgn}\;\left(x_{2}\right)),\\ {\dot{x}}_{2} & =x_{1}-x_{2},\\ {\dot{x}}_{3} & =-x_{2}, \end{array}$$

for which

$$A=\left(\begin{array}{ccc} 0 & -p & 0\\ 0 & 0 & 0\\ 0 & 0 & 0 \end{array}\right),\;g\left(x\right)=\left(\begin{array}{cc} & -px_{1}+px_{3}\\ & x_{1}-x_{2}\\ & -x_{2} \end{array}\right).$$

Other switch systems of form (1) are Sprott systems [11] or variants of the Shimizu–Morioka system [12].

Systems modeled by IVP (1) need not have classical solutions, as shown in the following example.

*Example 3.*  Consider, for example, the following discontinuous right-hand side equation:

$$\dot{x}=2-3\mathrm{sgn}\left(x\right)=\left\{\begin{array}{cc} 5,\; & x<0,\\ 2,\; & x=0,\\ -1,\; & x>0 \end{array}\right.\;x\left(0\right)=x_{0}.$$

Here,

g(x)=2,A=−3,s(x)=sgn(x).

For x(0)≠0, the equation has classical solutions

$$x\left(t\right)=\left\{\begin{array}{c} 5t+x_{0},\;x_{0}<0,\\ -t+x_{0},\;x_{0}>0. \end{array}\right.,\;x_{0}\in R.$$

**Figure 3:**
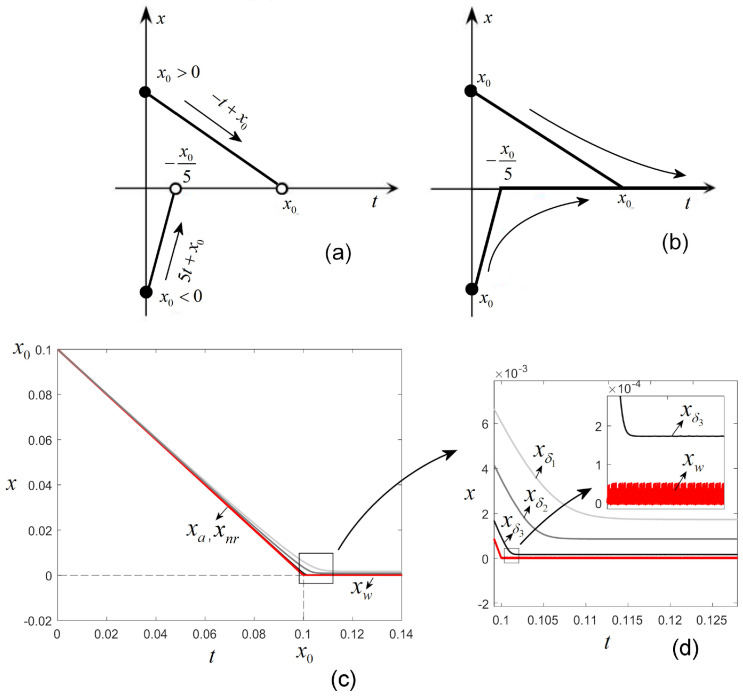
(**a**) Standard solutions of the discontinuous equation of Example 3; (**b**) generalized solutions; (**c**) numerical simulations; (**d**) zoomed-in detail. $x_{a}$ represents the (standard) analytical solution existing for $t\in [0,x_{0}=0.1]$; $x_{nr}$ represents the solution obtained with the ode45 procedure; $x_{w}$ represents the incorrect solution, the continuation of the $x_{nr}$ solution for $t>x_{0}$; $x_{\delta i}$, i=1,2,3, denotes the approximations for δ=1e−3,5e−4,1e−4, respectively.

The above two solutions tend to the axis x=0, which does not represent a classical solution to the problem since x=0 does not verify the equation, with the left-hand side being 0, while the right-hand side equals 2 (see Figure 3a).

Because IVP (1) might not have any solutions, another concept of solutions should be introduced.

## 3. Solutions to IVP (1)

To introduce a solution to IVP (1), the problem could be restated as an IVP with a differential inclusion (DI) and initial condition x(0):

(7) $$\dot{x}\in F\left(x\right),\;x\left(0\right)=x_{0},\;\mathrm{for}\;a.e.\;t\in I,$$

where $F:R^{n}⇉R^{n}$ is a set-valued function into the set of all subsets of $R^{n}$ that can be defined in several ways. (In (7), “for a.e. *t*” means except for a set *t* of measure 0). One of the widely used methods is Filippov regularization [6]:

(8) $$F\left(x\right)=\underset{\varepsilon >0}{⋂}\bar{conv(f(\{z\in R^{n}:∥z-x∥\leq \varepsilon \}\setminus M))}.$$

In (8), F(x) represents the convex hull of *f*, *M* is the null set of discontinuities of *f*, and ε is the radius of the ball centered in *x*. At point *x*, where *f* is continuous, F(x) is a point that coincides with the value of *f* at point F(x)=f(x), while at the points of discontinuity, F(x) has the value given by (7). Other regularization methods can be found, e.g., in [13] (see also [14]).

Note that by physical meanings, ε should be considered small enough so that the motion of the modeled physical system becomes close enough to a certain solution of DI (7).

By applying Filippov regularization to PWC IVP (1), one obtains the following IVP with DI:

(9) $$\dot{x}\in F\left(x\right):=f\left(x\right)+AS\left(x\right),\;x\left(0\right)=x_{0},$$

where *S* is the regularization of *s*. If $s\left(x\right)={\left(\mathrm{sgn}\;\left(x_{1}\right),\mathrm{sgn}\;\left(x_{2}\right),...,\mathrm{sgn}\;\left(x_{n}\right)\right)}^{T}$, the Filippov regularization of any $\mathrm{sgn}\;(x_{i})$ functions is

(10) $$\mathrm{Sgn}\left(x_{i}\right)=\left\{\begin{array}{cc} \{-1\}, & \;x_{i}<0,\\ {}[-1,1], & \;x_{i}=0,\\ \{+1\}, & \;x_{i}>0, \end{array}\right.$$

where i∈{1,2,...,n}. Here, the discontinuity set is M={0}.

For the preloaded compliance system, Filippov regularization refers only to $f_{2}$ at $x_{1}=0$, which can be considered a function of variables $X_{1}$ and $x_{2}$. The obtained set-valued function is

$$F_{2}\left(x_{1},x_{3}\right)=-2\gamma x_{1}-x_{1}-\mathrm{Sgn}\left(x_{1}\right)+p\cos\left(x_{3}\right),$$

with $\mathrm{Sgn}(x_{1})$ given by (10). The surface $F_{2}\left(x_{1},x_{3}\right)$ is presented in Figure 2b. The gray orthogonal surface actually only (defined for ε→0; see (8)) represents the set-valued function of $f_{2}$ and is a closed surface on the plane $x_{1}=0$, bounded by two sinusoidal curves, $p\cos(x_{3})$, shifted vertically one toward the other, with ±1 by the sgn function.

Therefore, problem (5) is transformed into the following DI:

$$\begin{array}{cc} {\dot{x}}_{1} & =x_{2},\\ {\dot{x}}_{2} & \in -2\gamma x_{1}-x_{1}-\mathrm{Sgn}\left(x_{1}\right)+p\cos\left(x_{3}\right),\\ {\dot{x}}_{3} & =\nu . \end{array}$$

**Remark 2.**  *As stipulated in [6], by embedding f into a set-value function F, which has enough regularity and is closely related to the trajectories of the underlying differential equation, we can stress the point that whenever f is continuous at x, the solution satisfies (1). Moreover, any classical solution to IVP (1) is a solution to IVP (9). Therefore, it is justified to call a solution of IVP (1) a solution of IVP (9).*

**Definition 3.**  *A* Filippov *(*generalized*)* solution *to the general set-valued IVP (7) is an absolutely continuous vector-valued function $x:I\to R^{n}$ satisfying (7) for a.e. t∈I.*

Some of the following results on existence and uniqueness can be found, in addition to the dedicated references, also in [15,16].

### 3.1. Existence

Denote with P the class of functions with the following *basic (Péano) conditions*: (a) *upper-semicontinuous* (USC) with (b) *non-empty*, (c) *closed*, and (d) *convex values*.

**Remark 4.**  *If f is locally bounded, the set-valued function F defined by the Filippov regularization is USC with non-empty, closed, and convex values, i.e., Péano’s conditions (compare [17] [Corollary 1, p.20] and [6] [Lemma 3, p.67]).*

On mild assumptions, IVP (7) has a generalized (Filippov) solution that could be a.e. unique. Because the set-valued function *F* enjoys enough regularity, the obtained d.i. may even have multiple solutions as well.

**Remark 5.**  *Because of the symmetric interpretation of a set-valued function as a graph, we can say that* a set-valued function satisfies a property if and only if its graph satisfies it*. For instance, a set-valued function is said to be closed if and only if its graph is closed [17,18]. Therefore, in practical problems, it is more convenient to consider the closure of the graph of F instead of the closed values of F.*

The main result of this subsection is the following.

**Theorem 6.**  *IVP (9) admits at least a generalized solution.*

**Proof.**  The function *S* being locally bounded verifies Péano’s conditions (Remark 4). With function *g* being Lipschitz continuous, the set-valued function F(x)=g(x)+AS(x) verifies Péano’s conditions and, therefore, verifies the existence of the result (Theorem 1, p.77, presented in [6]). □

*Example 4.*  The equation $\dot{x}=\mathrm{sgn}\;\left(x\right)$, where x(0)=0, has no classical solutions starting from x=0, but the regularized problem $\dot{x}\in \mathrm{Sgn}\left(x\right)$, where x(0)=0, admits multiple generalized solutions: x(t)=0 for $t<t_{*}$, and $x\left(t\right)=\pm \left(t-t_{*}\right)$ for $t>t_{*}$, where $t_{*}\geq 0$ could be *∞*.

This happens because in the ε-neighborhood of the discontinuity, the derivative can take an infinity of values of the set-valued function.

*Example 5.*  The equation $\dot{x}=-\mathrm{sgn}\;\left(x\right)$, where x(0)=0, does not admit solutions, while the associated DI $\dot{x}\in -\mathrm{Sgn}\left(x\right)$, where x(0)=0, admits a unique generalized solution $x\left(t\right)=t_{*}-t$ for $t\leq t_{*}$ and 0 for $t>t_{*}$.

Similarly, if $x\left(0\right)=x_{0}>0$, the equation in Example 3 has a positive generalized solution $x\left(t\right)=-t+x_{0}$ for $t<x_{0}$ and x(t)=0 for $t\geq x_{0}$. In other words, the solution can be now continuously prolonged once it crosses the axis x=0. Also, there is a unique negative solution for $x_{0}=x_{0}<0$, $x\left(t\right)=5t+x_{0}$ for $t<x_{0}$ and x(t)=0 for $t\geq x_{0}$ (Figure 3b).

### 3.2. Uniqueness

To prove the uniqueness of certain DIs, special Lipschitz conditions are required.

**Definition 7.**  *F satisfies a one-sided Lipschitz (OSL) condition with OSL constant λ if*

$$\langle \zeta^{\prime }-\zeta^{\prime \prime },x^{\prime }-x^{\prime \prime }\rangle \leq \lambda ∥x^{\prime }-x^{\prime \prime }∥,$$

*for all $\zeta^{\prime }\in F\left(x^{\prime }\right)$ and $\zeta^{\prime \prime }\in F\left(x^{\prime \prime }\right)$, with $x^{\prime },x^{\prime \prime }\in R^{n}$.*

The OSL condition is weaker than the classical Lipschitz condition or Lipschitz continuity. On the other hand, for explicit numerical methods for DIs and for higher-dimensional problems, the OSL condition is no longer adequate for uniqueness, and therefore, a stronger one-sided Lipschitz condition is required.

**Definition 8.**  *[3,4,8] The set-valued function F satisfies a strengthened one-sided Lipschitz (SOSL) condition if with OSL constants $\left(\lambda_{1},\lambda_{2},...,\lambda_{n}\right)$, the implication*

$$x_{i}^{\prime }>x_{i}^{\prime \prime }\Rightarrow \zeta_{i}^{\prime }\leq \zeta_{i}^{\prime \prime }+\lambda_{i}\left\|x^{\prime }-x^{\prime \prime }\right\|,$$

*is true for all $x^{\prime },x^{\prime \prime }\in R^{n}$, $\zeta_{i}^{\prime }\in F\left(x^{\prime }\right)$, and $\zeta_{i}^{\prime \prime }\in F\left(x^{\prime \prime }\right)$ and all components i=1,2,...,n.*

**Remark 9.**  *For n>1 the SOSL condition is stronger than the one-side lipschitz condition and weaker than the classical Lipschitz condition for single-valued right-hand sides. For n=1, the SOSL condition and OSL condition are equivalent [19].*

With the use of the uniqueness results in [19], the following result can be formulated.

**Lemma 10.**  *If the function g in IVP (9) is Lipschitz continuous and S satisfies the OSL condition, the set-valued function F satisfies the SOSL condition.*

In the particular case when function S(x)=−Sgn(x), the following useful result holds.

**Proposition 11.**  *The set-valued −Sgn satisfies the SOSL condition.*

**Proof.**  The set-value function Sgn is defined in R, and therefore, n=1. Consider first $x^{\prime }>x^{\prime \prime }$ for $x^{\prime },x^{\prime \prime }<0$. Then, $-\mathrm{Sgn}\left(x^{\prime }\right)=-\mathrm{Sgn}\left(x^{\prime \prime }\right)=1$, and the implication is verified. If $x^{\prime }<0$, $x^{\prime \prime }=0$, $-\mathrm{Sgn}(x^{\prime })=1$, and $max\{-\mathrm{Sgn}\left(x^{\prime \prime }\right)\}=1$, then the implication is verified again. Similarly, this holds for $0>x^{\prime }>x^{\prime \prime }$. □

The set-valued function +Sgn does not satisfy the SOSL condition.

**Theorem 12.**  *[19] If F verifies the SOSL condition, then the IVP admits at most one generalized solution.*

The main result in this subsection is the following (see also [19]).

**Theorem 13.**  *If the matrix is negative in IVP (9), the IVP admits a unique solution.*

**Proof.**  IF the elements of *A* are all negative, then AS satisfies the SOSL, and the problem admits at most one solution (Theorem 12). On the other hand, because *F* verifies Péeano’s conditions (Remark 4), the IVP admits at least one solution. □

Here, uniqueness means that once the solution arrives on the discontinuity surface (or on an intersection of such surfaces), it can be uniquely continued in the positive time direction. At the same time, note that uniqueness, in the positive time direction, does not necessarily imply uniqueness in the negative time direction.

The SOSL condition is only sufficient and not necessary. However, compared with the classical Lipschitz condition, the SOSL condition is much easier to apply directly in examples. Therefore, due to the negativeness of *A* in Examples 1, 2, 3, and 5 the underlying equations admit a unique generalized solution.

### 3.3. Approximate Selection

There exist several numerical schemes to integrate DIs to numerically find the generalized solutions (see, e.g., [19,20,21]).

Let *N* be some natural number $N\in N^{\prime }\subset N$, with N being a subsequence of N tending to infinity, h=T/N, and an equidistant grid

$$t_{0}=0<t_{1}<...<t_{N}=T$$

Let a sequence of discrete-time inclusions be associated with IVP (7) in the following form:

(11) $$y_{k+1}\in G_{k}^{N}\left(h;y_{k}\right),k=0,1,...,N-1,y_{0}=x_{0},$$

where $G_{k}^{N}:R^{r}⇉R^{n}$ is a discrete-time set-valued function. Then, a solution of (11) on I is any sequence of N+1 vectors $y_{0},y_{1},...,y_{N}$ that satisfies (11) for k=0,1,...,N−1.

With these ingredients, the simplest explicit difference scheme for DIs, which is the set-valued version of the Euler discretization for differential equations, has the form

(12) $$G_{k}^{N}=\left(h;y_{k}\right)=y_{k}+hF\left(t_{k},y_{k}\right).$$

The simplest way to implement scheme (12) is to randomly choose a value of *F* when the solution crosses the discontinuity surface. This means that once the solution enters within a small enough ε-neighborhood of the discontinuity surface, one chooses a random value of the set-value function *F* (a segment in the uni-dimensional case).

The convergence of Euler schemes (11) and (12) is presented in various forms in, e.g., [6] [Theorem 1, p.77], [17] [Theorem 3, p.98], and [18] [Theorem 10.1.3, p.390].

Despite the fact that there exist other numerical methods to solve DIs (see, e.g., [5,8,20] and so on), a simpler way considered in this paper is to convert the DI into a continuous single-valued problem via approximate results offered by the theory of DIs, after which the problem can be treated numerically as a simple system of continuous ODEs.

**Definition 14.**  *The function $f:R^{n}\to R^{n}$ is a* selection *(approximate) of a set-valued function F if f(x)∈F(x) for every $x\in R^{n}$ [17,18].*

Generally, a set-valued function admits infinitely many selections, a fact that represents a major advantage in approaching discontinuous systems coming from practical problems. The selections can be discontinuous with respect to the state variable or time. In this paper, continuous selections are considered.

The set-valued function defined with Fillipov’s regularization verifies the following result ([17] [Theorem 4, p.101], [18] [Theorem 9.2.1, p.358]).

**Theorem 15**  (Cellina Theorem). *Let a USC set-valued function $F:R^{n}⇉R^{n}$ with non-empty convex values. Then, for every ε>0, there exists a locally Lipschitz selection $f_{\varepsilon }:R^{n}⇉R^{n}$ such that*

$$Graph\left(f_{\varepsilon }\right)\subset Graph\left(F\right)+\varepsilon B$$

*which is a sphere of radius ε.*

The proof of Theorem 15 is constructive and therefore useful to use in applications.

Let S(x)=Sgn(x). An approximate function for Sgn(x) is

(13) $${\mathrm{Sgn}}_{\varepsilon }\left(x\right)=\frac{2}{\pi }\arctan\frac{x}{\delta }$$

where ε represents the radius of the sphere centered at *x*, and δ is a relatively small positive number that controls the slope in the ε-neighborhood of the discontinuity. Obviously, between ε and δ, there exists a direct connection: at low values of ε, there are low values of δ [16].

Other used approximate functions are $\frac{x}{\sqrt{x^{2}+\delta }},\;\frac{2}{1+e^{-\frac{x}{\delta }}}-1$, e.t.c.

**Figure 4:**
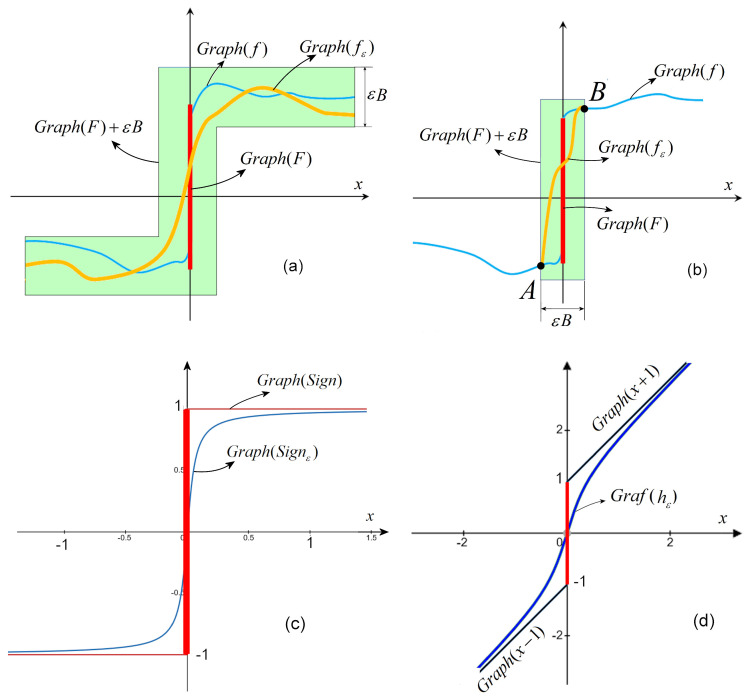
(**a**) Sketch of the approximate selection of *F*; (**b**) locally approximate selection of *F*. Points *A* and *B* represent the continuous connections of the selection with *f*. (**c**) The sigmoid ${\mathrm{Sgn}}_{\varepsilon }$ approximation of the set-valued function Sgn; (**d**) graph of the approximation of *h* in Example 1.

The approximate function, ${\mathrm{Sgn}}_{\varepsilon }$, is presented in Figure 4c, where the graph is plotted for a large value of δ, $\delta =10^{-1}$, for a clear image, and the approximate function of *h* in Example 1, $h_{\varepsilon }$, is drawn in Figure 4d.

Note that, as stated by Theorem 15, the selection $f_{\varepsilon }$ approximates *f* (g+AS in our case) along its entire domain R (see the sketch in Figure 4a). However, in [16], it is shown how the approximation could be improved by considering only a small neighborhood of *S* (see Figure 4b). In this way, the approximation errors are reduced.

## 4. Numerical Simulations

Classical numerical schemes with which to integrate ODEs, such as Runge–Kutta, Euler, and so on, and also their implemented variants in software like Matlab (such as ode45) or Mathematica (such as ndsolve), are deduced for continuous ODEs. However, they can be used to integrate discontinuous problems like those presented in this paper without considering the discontinuities, which is a fact that could lead to incorrect results. For example, consider Example 4 and integrate it with Matlab procedure ode45 on t∈[0,0.13] (Figure 3c,d) without taking into account the discontinuity and also by considering the approximation of the equation for the same initial condition $x_{0}=0.1$. As can be seen in Figure 3c, the analytical solution, denoted as $x_{a}$ (red plot), is very well indistinguishable here from the numerically determined solution, denoted as $x_{nr}$, within the interval $\left[0,x_{0}\right]$. After $t=x_{0}$, the analytical solution no longer exists, as proved in Example 4. However, the numerical integration keeps going for any chosen length of time by giving an incorrect result (denoted as $x_{w}$). Moreover, as can be seen in the zoom-in in Figure 3d, the numerical integration without considering the discontinuity problem leads to a possible wrong interpretation of the result: for example, for $t>x_{0}=0.1$, the trajectory would be chaotic even if it is not. The integration of the approximated equation for three values of parameter δ, $10^{-3},5\times 10^{-4}$, and $10^{-4}$ (gray plots denote solutions $x_{\delta_{1}},x_{\delta_{2}}$, and $x_{\delta_{3}}$ in the successive zoom-ins in Figure 3d), unveils the fact that decreasing δ, the (correct) solution, increasingly tends toward the axis x=0 for all t≥0. However, solutions will only be asymptotically identical to the axis, compared with the local approximation presented in [16].

**Figure 5:**
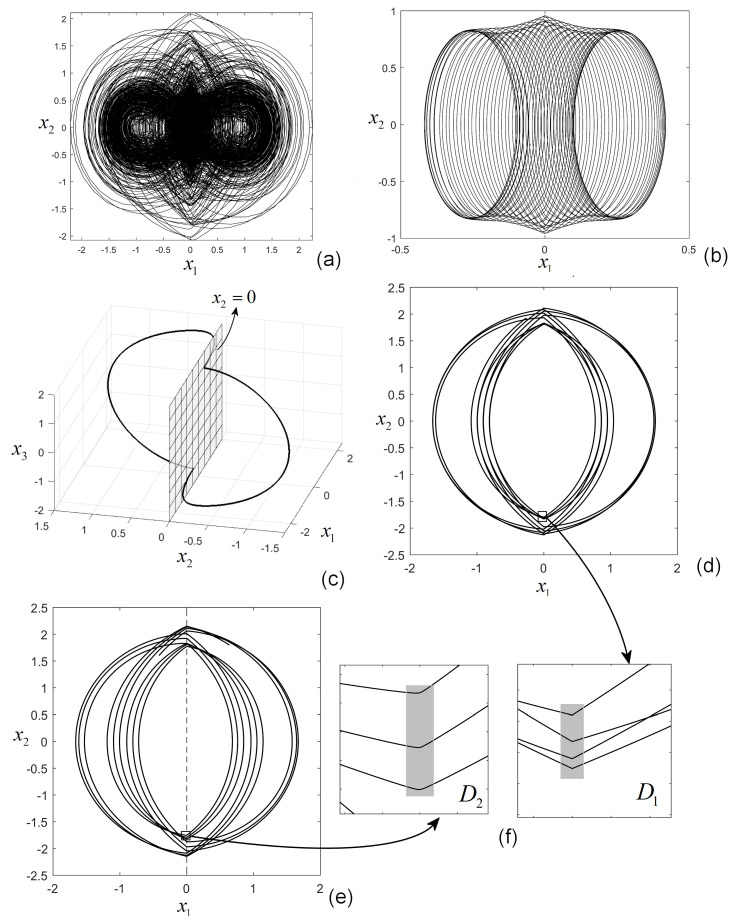
(**a**) Chaotic trajectory of preloaded compliance system (2); (**b**) quasiperiodic trajectory of the preloaded compliance system; (**c**) stable periodic trajectory of steam turbine control system (6); (**d**,**e**) two spirals of the chaotic trajectory in (**a**) for the same initial condition and parameters but without and with continuous approximation; (**f**) zoomed-in details $D_{1}$ and $D_{2}$ revealing the effect of the approximation.

After applying the algorithm of continuous approximation presented in Section 3 to Examples 1 and 2, some trajectories are presented in Figure 5. In Figure 5a,b, a chaotic trajectory and a quasiperiodic trajectory of the preloaded compliance system, respectively, are presented, while in Figure 5c, a stable cycle of the system in Example 2 is presented. As is typical for discontinuous systems, at the points where the trajectory crosses the discontinuity surface, due to the different non-smoothness quality of the trajectory on the two sides of the surface, the trajectory can change the directions and “corners” can appear: axis Ox1, $x_{1}=0$, in Figure 5a, and the plane $x_{2}=0$ for the steam turbine in Figure 5c. However, because of the continuous approximation, these corners are smoothed. Figure 5d,e present comparatively few spirals from the chaotic trajectory in Figure 5a without and with approximation, respectively. The corners can be viewed in zoom-ins $D_{1}$ and $D_{2}$ in Figure 5f.

## 5. Conclusions

In this paper, the existence and uniqueness of solutions for a class of switch dynamical systems are studied. As a novelty, it is shown how the initial discontinuous problem can be transformed into a continuous one using Filippov’s regularization. Due to the regularity properties of the obtained differential inclusion, where the right-hand side is a set-valued function, the approximate selection theory can be applied (Cellina’s Theorem 15), with which the differential inclusion is restarted as a continuous single-valued problem. Compared to other existing approaches, where the discontinuity is managed by directly replacing the discontinuity function with a continuous one, without analytical proof, in this paper, we show why this approximation can be achieved. Also, a simple sufficient uniqueness criterion, the SOSL condition, is proposed to deduce the uniqueness: the negativeness of the elements of matrix *A*. Moreover, it is numerically shown that applying the standard numerical schemes for differential equations without considering the discontinuity could lead to incorrect solutions. To avoid the wrong result, this work proposes a continuous approximation algorithm. Among some standard (theoretical) examples, a preloaded compliance mechanical system is considered. As a future research direction, the experimental implementation of the proposed continuous approximation in systems with discontinuity (such as, e.g., an oscillator) could offer advantages by comparison.

## Data Availability

No new data were created or analyzed in this study.
